# High-Throughput Screening Identifies Genes Required for *Candida albicans* Induction of Macrophage Pyroptosis

**DOI:** 10.1128/mBio.01581-18

**Published:** 2018-08-21

**Authors:** Teresa R. O’Meara, Kwamaa Duah, Cynthia X. Guo, Michelle E. Maxson, Ryan G. Gaudet, Kristy Koselny, Melanie Wellington, Michael E. Powers, Jessie MacAlpine, Matthew J. O’Meara, Amanda O. Veri, Sergio Grinstein, Suzanne M. Noble, Damian Krysan, Scott D. Gray-Owen, Leah E. Cowen

**Affiliations:** a Department of Molecular Genetics, University of Toronto, Toronto, Ontario, Canada; b Program in Cell Biology, Hospital for Sick Children, Toronto, Ontario, Canada; c Department of Pediatrics, University of Rochester School of Medicine and Dentistry, Rochester, New York, USA; d Division of Pediatric Infectious Disease, University of Iowa, Iowa City, Iowa, USA; e Department of Microbiology and Immunology, University of California, San Francisco, San Francisco, California, USA; f Department of Pharmaceutical Chemistry, University of California, San Francisco, San Francisco, California, USA; g Department of Biochemistry, University of Toronto, Toronto, Ontario, Canada; University of British Columbia

**Keywords:** *Candida*, cell wall remodelling, functional genomics, fungal morphogenesis, fungal pathogenesis, host-pathogen interaction, pyroptosis

## Abstract

The innate immune system is the first line of defense against invasive fungal infections. As a consequence, many successful fungal pathogens have evolved elegant strategies to interact with host immune cells. For example, Candida albicans undergoes a morphogenetic switch coupled to cell wall remodeling upon phagocytosis by macrophages and then induces macrophage pyroptosis, an inflammatory cell death program. To elucidate the genetic circuitry through which C. albicans orchestrates this host response, we performed the first large-scale analysis of C. albicans interactions with mammalian immune cells. We identified 98 C. albicans genes that enable macrophage pyroptosis without influencing fungal cell morphology in the macrophage, including specific determinants of cell wall biogenesis and the Hog1 signaling cascade. Using these mutated genes, we discovered that defects in the activation of pyroptosis affect immune cell recruitment during infection. Examining host circuitry required for pyroptosis in response to C. albicans infection, we discovered that inflammasome priming and activation can be decoupled. Finally, we observed that apoptosis-associated speck-like protein containing a CARD (ASC) oligomerization can occur prior to phagolysosomal rupture by C. albicans hyphae, demonstrating that phagolysosomal rupture is not the inflammasome activating signal. Taking the data together, this work defines genes that enable fungal cell wall remodeling and activation of macrophage pyroptosis independently of effects on morphogenesis and identifies macrophage signaling components that are required for pyroptosis in response to C. albicans infection.

## IMPORTANCE

Candida albicans is a natural member of the human mucosal microbiota that can also cause superficial infections and life-threatening systemic infections, both of which are characterized by inflammation. Host defense relies mainly on the ingestion and destruction of C. albicans by innate immune cells, such as macrophages and neutrophils. Although some C. albicans cells are killed by macrophages, most undergo a morphological change and escape by inducing macrophage pyroptosis. Here, we investigated the C. albicans genes and host factors that promote macrophage pyroptosis in response to intracellular fungi. This work provides a foundation for understanding how host immune cells interact with C. albicans and may lead to effective strategies to modulate inflammation induced by fungal infections.

## INTRODUCTION

Professional phagocytes, including macrophages and neutrophils, provide the front-line host response to invading microorganisms (1). Macrophages readily engulf diverse microbes, including fungi, by recognizing pathogen-associated molecular patterns (PAMPs), such as β-glucan (2). For Candida albicans, a leading fungal human pathogen, engulfment by macrophages triggers a morphological transition from the yeast form to filamentous cells. The capacity to transition between yeasts and filaments is strongly correlated with virulence, and mutants that are unable to undergo this transition are typically attenuated in virulence, although there are some exceptions (3–5).

This C. albicans filamentation in macrophages was previously thought to be coupled to the induction of macrophage pyroptosis, an inflammatory host cell death program mediated via activation of the NLRP3 inflammasome and caspase-1 (6–9). Pyroptosis occurs early in response to C. albicans, between 3 and 6 h after phagocytosis, after which a secondary form of macrophage cell death occurs (10, 11). Pyroptosis in response to fungi is a broadly conserved host response, as the evolutionarily distant fungal human pathogens Cryptococcus neoformans, Paracoccidioides brasiliensis, and Histoplasma capsulatum can also induce macrophage pyroptosis (12–14). The importance of the inflammasome in host antifungal defense is demonstrated by the hypersusceptibility of NLRP3-deficient mice to systemic fungal infection (12, 15–18). Inflammasome activation and pyroptosis trigger the release of interleukin-1β (IL-1β), a cytokine that acts both locally and systemically to recruit polymorphonuclear leukocytes (PMNs) to sites of infection, a process crucial for defense against fungal pathogens (19). In other contexts, inflammasome activation can be detrimental to host outcomes. For example, one of the strongest host signatures during C. albicans infection of the vulvovaginal tract is NLRP3 inflammasome activation (20), and this is a major driver of immunopathology (20–22). This highlights the differential requirements and utility of inflammasome activity at different sites of infection in the host and the delicate balance of immune responses required to successfully combat fungal infections.

The relationship between fungal morphogenesis, cell wall remodeling, and activation of macrophage pyroptosis is also complex. Although most C. albicans mutants with defects in filamentation are also unable to induce macrophage pyroptosis, it is clear that filamentation is neither necessary nor sufficient for activation of pyroptosis (23, 24). We and others recently discovered that it is not filamentation *per se* that activates pyroptosis but rather a coregulated process of fungal cell wall remodeling in response to the macrophage environment (10, 23, 24). The C. albicans cell wall is composed of an outer layer of mannan and an inner layer of chitin and (1–3)-β-linked glucan with long (1–6)-β-linked side chains (25, 26). Previous work implicated glucan exposure in pyroptosis (10, 27, 28), and we demonstrated that, upon phagocytosis, C. albicans undergoes the active cell wall remodeling that is required for the induction of pyroptosis (24). This cell wall remodeling involves exposure of specific mannose-containing moieties that are sufficient to activate macrophage pyroptosis without requiring fungal cell viability (24). However, the genetic circuitry through which fungi orchestrate this inflammatory host cell death program remained unknown.

Functional genomic and genetic analyses provide a powerful strategy to identify genes that govern key interactions at the host-pathogen interface. Here, we use high-throughput screening of a tetracycline-repressible conditional expression strain collection with coverage of 40% of the C. albicans genome to identify genes that influence C. albicans interaction with host immune cells. As a primary approach, we perform imaging-based arrayed screens to systematically assess the capacity of each gene replacement and conditional expression (GRACE) strain to induce host cell lysis upon transcriptional repression of target genes with the tetracycline analogue doxycycline (DOX). Through this systematic analysis, we identified 98 genes that are dispensable for filamentation in macrophages but required for robust activation of pyroptosis. Detailed analysis of fungal genes required for pyroptosis highlights a role for the glycosylphosphatidylinositol (GPI)-anchored Pga52 protein in driving macrophage responses. We demonstrate that genes encoding each component of the Hog1 cascade are required for pyroptosis and that this cascade transcriptionally regulates cell wall remodeling within the macrophage. Defects in remodeling the fungal cell wall are associated with defects in inflammasome activation but not priming. In our analysis of host factors that drive pyroptosis in response to C. albicans, we observed that macrophages require both Toll-like receptor (TLR) and C-type lectin receptor (CLR) signaling to prime the inflammasome in response to C. albicans infection and that apoptosis-associated speck-like protein containing a CARD (ASC) oligomerization, a marker of inflammasome activation, does not depend upon phagolysosomal rupture. Our findings establish specificity in the genetic circuitry that enables C. albicans activation of host programed cell death and highlight host signaling components required for alerting the cytosolic surveillance system to fungal capture.

## RESULTS

### High-throughput screening of C. albicans-macrophage interactions.

**FIG 1:**
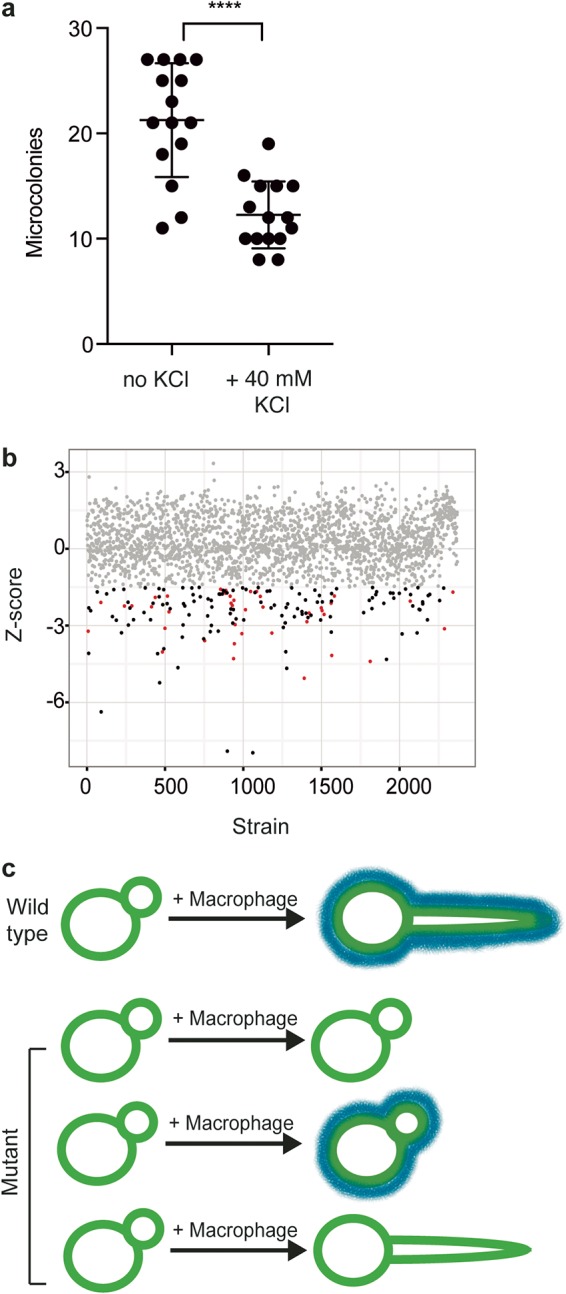
High-throughput screening to examine C. albicans*-*macrophage interactions. (a) Macrophage pyroptosis increases fungal survival. Macrophages were incubated with diluted C. albicans cells in the presence or absence of 40 mM KCl for 24 h. Microcolonies were counted from two biological replicates, with 8 technical replicates. ****, *P* < 0.0001 (unpaired *t* test). Error bars represent standard deviations. (b) Screening approach. J774A.1 macrophages were coincubated for 3 h with GRACE strains in the presence of 0.05 µg/ml DOX to repress target gene expression. Lysis events were determined by counting the number of propidium iodide foci. To detect whether a mutant has a significant defect in macrophage pyroptosis, we developed a model for host cell lysis in response to the wild-type strain using the locally weighted scatterplot smoothing method (LOESS) and evaluated each mutant relative to the model. Each dot represents an individual GRACE strain, with the calculated *Z* score shown. The grey dots represent strains with no significant difference from the wild-type results. Red dots represent strains with a significant decrease in induction of host cell lysis rates and a defect in filamentation. Black dots represent strains with a significant decrease in host cell lysis rates and no defect in filamentation. (c) Model for C. albicans interactions with macrophages. Blue lines represent cell surface moieties that are required for pyroptosis.

Macrophages are able to kill C. albicans, but C. albicans can also induce macrophage pyroptosis, resulting in the death of the host cell. To examine the impact of pyroptosis on the survival of C. albicans cells, we performed *in vitro* infections of macrophages with C. albicans in the presence or absence of potassium to inhibit pyroptosis (29) and measured the numbers of CFUs after 24 h of coincubation. Addition of 40 nM potassium did not inhibit fungal growth and was sufficient to block pyroptosis (see Fig. S1a in the supplemental material). We observed a decrease in fungal CFU levels when pyroptosis was inhibited (Fig. 1a), suggesting that fungi can use pyroptosis to evade killing by macrophages.

10.1128/mBio.01581-18.1 FIG S1  (a) Potassium chloride does not affect C. albicans survival but does inhibit ASC speck formation. C. albicans cells were incubated with 40 nM KCl at 37°C with 5% CO_2_ for 24 h. Microcolony counts or ASC speck formation was determined from 8 technical replicates. Data represent results from one of two biological replicates. Error bars represent standard deviations. (b) Variation of wild-type models for each macrophage batch. Each wild-type model is fitted by calling the stats::loess [formula = log (PI.counts) ~ OD, data=wt] function in R, with the shaded region representing the 95% confidence interval under an assumed t-distribution of errors and estimated effective degrees of freedom. (c) For a secondary reporter for NLRP3-dependent pyroptosis, we used murine macrophages that harbor an ASC-mCherry fluorescent protein reporter that oligomerizes upon NLRP3 activation. The macrophages were infected with the *tetO-MNN24*/*mnn24*Δ strain in the presence or absence of 0.5 µg/ml DOX. This strain is able to filament but is not capable of inducing pyroptosis. Bar, 20 µm. Download FIG S1, TIF file, 1.2 MB. Copyright © 2018 O’Meara et al. 2018 O’Meara et al. This content is distributed under the terms of the Creative Commons Attribution 4.0 International license.

To investigate the genetic circuitry governing the interactions between C. albicans and macrophages, we developed a high-throughput imaging-based screen to identify C. albicans genes required for induction of macrophage cell death. The C. albicans GRACE collection of 2,356 tetracycline-repressible conditional expression strains (24, 30) was cocultured with J774A macrophages in the presence or absence of 0.05 µg/ml doxycycline (DOX). This concentration of DOX has previously been demonstrated to repress gene expression in this system (24). After 3 h of coincubation, staining with propidium iodide (PI) was used to estimate the rate of macrophage cell death. For each batch of macrophages, each mutant was evaluated relative to a wild-type control, using the log of propidium iodide (PI) counts as a function of optical density at 600 nm (OD_600_) and the locally weighted scatterplot smoothing method (LOESS) (Fig.  1b; see also Fig.  S1).

A key aspect of the interaction between C. albicans and macrophages is the morphological transition from yeast to filamentous growth. Although most C. albicans mutants that are defective in filamentation are unable to induce macrophage pyroptosis, filamentation is neither necessary nor sufficient for activation of pyroptosis (23, 24). To identify the subset of C. albicans factors that are required specifically for activation of macrophage cell death (and not filamentation), we also determined the morphology of each C. albicans GRACE strain during macrophage infection using high-throughput imaging. This allowed categorization of mutants into those that affect pyroptosis only, both pyroptosis and morphogenesis, or morphogenesis only (Fig. 1c).

### Diverse fungal genes are required for inducing macrophage pyroptosis.

**FIG 2:**
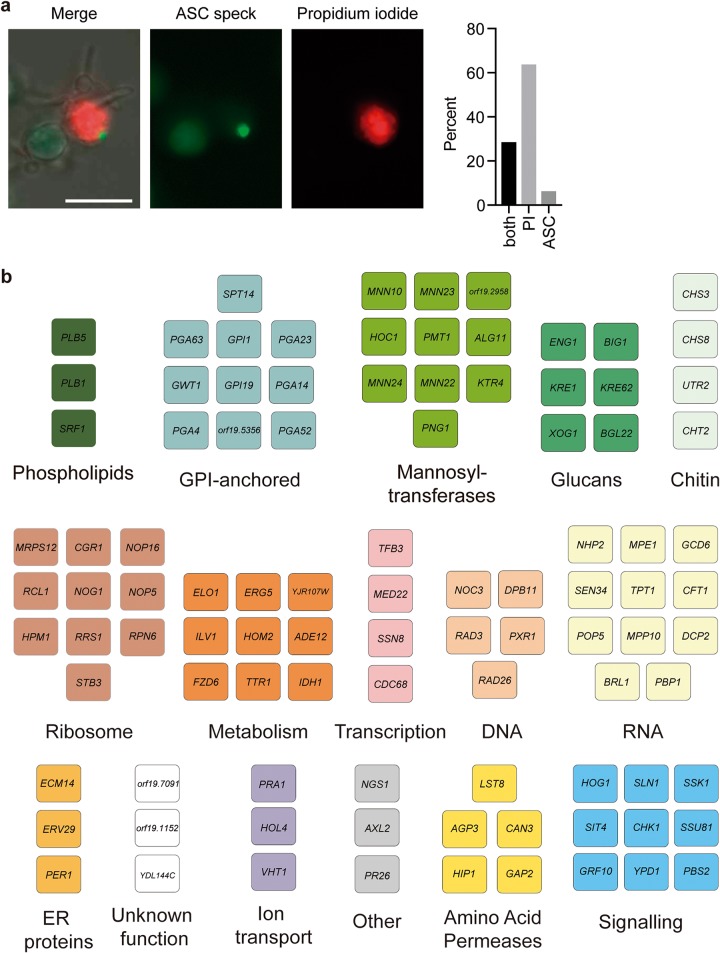
High-throughput screening identifies filamentation-competent mutants that are defective in activation of macrophage pyroptosis. (a) Images showing ASC-cerulean macrophages infected with C. albicans and stained with propidium iodide. Bar, 20 µm. The bar chart shows the percentage of cells positive for ASC specks only, propidium iodide staining only, or both. (b) We identified 98 genes that are required for wild-type levels of pyroptosis but that have no effect on filamentation. Functional clusters were defined by Gene Ontology (GO) term annotation. ER, endoplasmic reticulum.

Host cell death in response to C. albicans can occur by multiple mechanisms. One of these is pyroptosis, which is dependent upon ASC oligomerization, the NLRP3 inflammasome, and caspase-1 (6–9). To validate the screening results and examine the relationship between PI staining and the pyroptotic cell death program, we used a reporter system for inflammasome activation. We infected murine macrophages that harbor an ASC-fluorescent protein (FP) reporter and determined the number of ASC-FP paranuclear protein specks after 4 h of coincubation. By counting only infected macrophages, we normalized the pyroptosis rates to those of macrophages that contained C. albicans. The ASC specks are indicative of ASC oligomerization and activation of the inflammasome (31, 32). We observed that ASC speck formation tracks well with PI staining (Fig. 2a), although there are other additional mechanisms that can lead to host cell death. For each mutant identified in the screen, we assessed ASC speck formation and filamentation by microscopy.

Given that phagocytosis of C. albicans normally leads to simultaneous filamentation, cell wall remodeling, and exposure of the trigger of pyroptosis, most mutants with defects in filamentation are also defective in pyroptosis (10, 23, 24, 27, 33). By focusing on mutants that retain the ability to filament in macrophages while failing to induce pyroptosis, we sought to identify genes required specifically for induction of the host pyroptotic cell death program. From this analysis, we identified 67 fungal strains that failed to induce ASC specks while forming normal filaments in macrophages (Fig. 2b; see also Table S1 in the supplemental material). This greatly expands the previously known repertoire of fungal genes that modulate macrophage pyroptosis and uncouples the genetic control of filamentation from activation of macrophage pyroptosis.

10.1128/mBio.01581-18.7 TABLE S1  Filament-competent but pyroptosis defective strains. Download TABLE S1, XLSX file, 0.07 MB. Copyright © 2018 O’Meara et al. 2018 O’Meara et al. This content is distributed under the terms of the Creative Commons Attribution 4.0 International license.

### Specific cell wall biosynthetic genes are required for induction of pyroptosis.

From our initial analysis, we identified multiple cell wall genes that are necessary for pyroptosis. To test the hypothesis that any alterations in cell wall architecture would have similar results, we examined GRACE strains with alterations in the expression levels of 106 additional genes with known or predicted roles in cell wall remodeling that were not identified by our initial high-throughput screen. The strains were evaluated for the capacity to induce pyroptosis using the ASC reporter macrophage cell line and a higher level of DOX (0.5 µg/ml) to more strongly repress target gene expression, as 0.05 µg/ml DOX may not be sufficient for some genes. As expected, many of the strains exhibited defects in filamentation (42 strains; Table S2). Among the strains that were able to filament in macrophages, we identified 31 additional genes required for inducing pyroptosis and 65 genes with no effect on ASC speck formation (Table S2). This implicates a specific remodeling of the fungal cell surface in triggering this host cell death program rather than a general defect in cell wall composition.

10.1128/mBio.01581-18.8 TABLE S2  Role of cell wall genes in macrophage infections. Download TABLE S2, XLSX file, 0.07 MB. Copyright © 2018 O’Meara et al. 2018 O’Meara et al. This content is distributed under the terms of the Creative Commons Attribution 4.0 International license.

**FIG 3:**
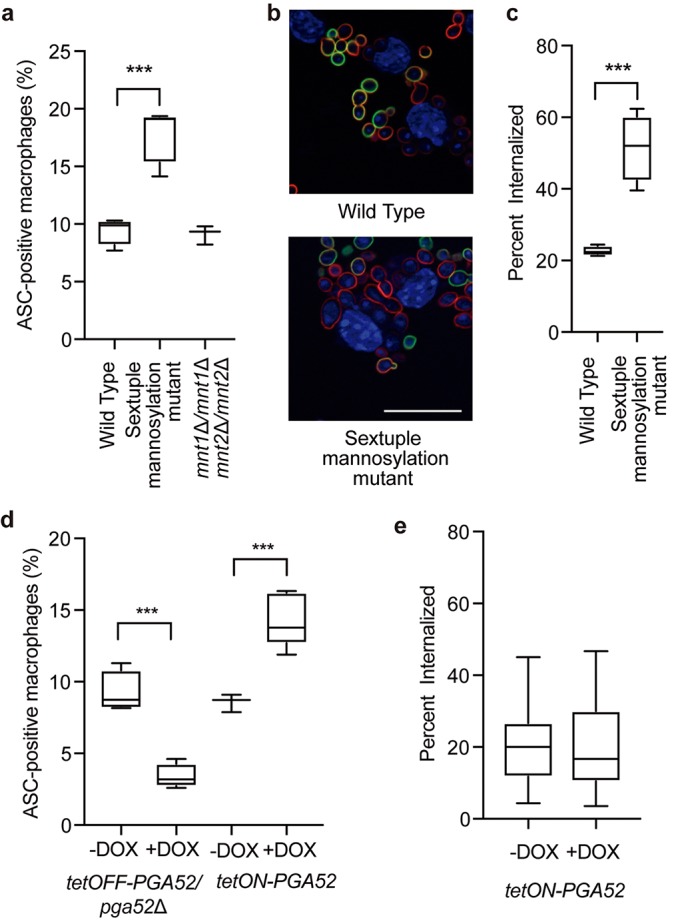
Defects in cell wall affect pyroptosis and phagocytosis. (a) Levels of ASC speck formation in response to mannosylation mutants. The sextuple mannosylation mutant has deletions in the *MNN2*, *MNN24*, *MNN22*, *MNN23*, *MNN26*, and *MNN21* genes. Macrophages were infected at an MOI of 1:3. ASC specks were quantified in ASC-mCherry macrophages after 4 h of infection in at least two biological replicates. ***, *P* < 0.0005 (1-way ANOVA). Error bars represent standard deviations. (b) The sextuple mannosylation mutant has increased phagocytosis. Cells were visualized by staining for DAPI (blue channel). Extracellular fungi are stained in green, and total fungi are stained in red. Bar, 20 µm. (c) The sextuple mannosylation mutant has increased rates of internalization. Internalization was measured after 30 min of coincubation. The numbers of internalized cells were determined from two biological replicates and plotted as the average rate of internalization from at least 4 fields of view. ***, *P* < 0.0005 (1-way ANOVA). Error bars represent standard deviations. (d) Levels of ASC speck formation for *PGA52* mutants. GRACE tetracycline-repressible strains were incubated with or without 0.5 µg/ml DOX overnight and during infection. Tetracycline-inducible strains were incubated overnight in 50 µg/ml DOX and 10 µg/ml DOX during infection. Macrophages were infected at an MOI of 1:3. ASC specks were calculated in ASC-mCherry macrophages after 4 h of infection in at least two biological replicates. ***, *P* < 0.0005 (unpaired *t* tests). Error bars represent standard deviations. (e) Overexpression of *PGA52* does not influence phagocytosis rates. The *PGA52* tetracycline-inducible strain was incubated overnight in 50 µg/ml DOX and 10 µg/ml DOX during infection. Internalization was measured after 30 min of coincubation. The numbers of internalized cells were determined from two biological replicates and plotted as the average rate of internalization from at least 10 fields of view. Error bars represent standard deviations. Significance was determined by 1-way ANOVAs.

In previous work, we demonstrated that mannosylated cell wall components are required for previously phagocytized and then heat-killed C. albicans to induce pyroptosis. Consistent with those results, we observed that multiple mannosyltransferases are required for pyroptosis (Fig. 2). We saw a potential signature for those proteins involved in extending the branches on N-linked mannosyl groups, such as Mnn10, Mnn22, Mnn23, and Mnn24. We then investigated the role of mannose in more detail using higher-order mannosyltransferase mutants (34), which allowed us to investigate genes with redundant functions. Consistent with our results obtained with the GRACE strains, deletion of genes encoding the Mnt1 and Mnt2 α-1,2 mannosyltransferases had no effect on pyroptosis, even in a double mutant strain (Fig. 3a), suggesting that O-linked mannosylation is not required for inducing pyroptosis. Surprisingly, we observed that deletion of six N-linked mannosyltransferase genes (*MNN2*, *MNN24*, *MNN22*, *MNN23*, *MNN26*, and *MNN21*) (34) significantly increased pyroptosis levels (Fig. 3a). However, we also observed a significant increase in levels of phagocytosis for this strain (Fig. 3b and c). Therefore, we hypothesize that the elevated level of phagocytosis may drive increased pyroptosis, bypassing the requirement for these specific mannose genes.

An additional signature in our screen was a requirement for multiple GPI-anchored proteins, many of which do not have an annotated function in C. albicans. GPI-anchored proteins can modulate cell wall architecture and are often highly mannosylated (35). To test whether overexpression of GPI-anchored proteins can stimulate pyroptosis, we tested four strains with different GPI-anchored proteins under the control of a DOX-inducible promoter (36) and monitored ASC speck formation. Overexpression of three of the GPI-anchored protein-encoding genes or the *LEU3* control gene had no significant effect on ASC speck formation (Fig. S2b). However, overexpression of *PGA52* was sufficient to cause a significant increase in ASC speck formation compared with the no-DOX control at the 4-h time point (Fig. 3d; see also Fig. S2a). Moreover, there was no change in the phagocytosis rate of the *PGA52* overexpression strain (Fig. 3e), suggesting that, unlike the sextuple mannose mutant results, the increase in ASC speck formation in response to *PGA52* overexpression cannot be attributed to an increase in phagocytosis. We then examined the impact of *PGA52* depletion on survival in macrophages by measuring CFU levels after 24 h of infection and observed a decrease in the CFU levels compared with the no-DOX control (Fig. S2c), similarly to the results obtained by chemically inhibiting pyroptosis with potassium chloride.

10.1128/mBio.01581-18.2 FIG S2  (a) Induction of *PGA52* overexpression with doxycycline is significantly different from the results seen with a control strain. Error bars represent standard errors of the means. (b) Overexpression of other GPI-anchored proteins does not affect ASC-speck formation. Each strain was incubated overnight in 50 µg/ml DOX to induce overexpression, and with 10 µg/ml DOX during infection. ASC-mCherry macrophages were incubated with the indicated strains at an MOI of 1:3 for 4 h before imaging. Pyroptosis was quantified from two technical replicates from at least two biological replicates. Error bars represent standard deviations. Significance was determined by 1-way ANOVA. (c) Depletion of *PGA52* results in decreased survival in macrophages. C. albicans cells were incubated with or without 0.5 µg/ml DOX at 37°C with 5% CO_2_ for 24 h. Microcolony counts were determined from 8 technical replicates. Percent survival was calculated compared with the no-DOX samples. Data represent results from one of two biological replicates. Error bars represent standard deviations. (d) Depletion of *PGA52* has no significant effect on drug sensitivity. MIC assays were performed in YPD medium at 30°C for 48 h, and optical densities at 600 nm were averaged for two biological replicates, with two technical replicates each. Percent growth is normalized to the no drug condition. To deplete target gene expression, the strains were incubated in 0.5 µg/ml doxycycline (DOX). Download FIG S2, TIF file, 1.5 MB. Copyright © 2018 O’Meara et al. 2018 O’Meara et al. This content is distributed under the terms of the Creative Commons Attribution 4.0 International license.

Pga52 is a GPI-anchored protein with no annotated function, although it is transcriptionally induced in macrophages and exposed on hyphal cell surfaces (37, 38). We tested whether Pga52 plays a role in cell wall integrity in C. albicans using MIC assays with cell wall- and cell membrane-perturbing agents. There was no significant difference in the levels of sensitivity to calcofluor white, caspofungin, or fluconazole in the Pga52 depletion strains (Fig. S2d), suggesting that Pga52 does not play a major role in cell wall integrity. Instead, this protein appears to modulate interactions with the host. Further studies will be necessary to understand the function of Pga52 and how it contributes to C. albicans interactions with host cells.

### The Hog1 cascade regulates macrophage pyroptosis.

**FIG 4:**
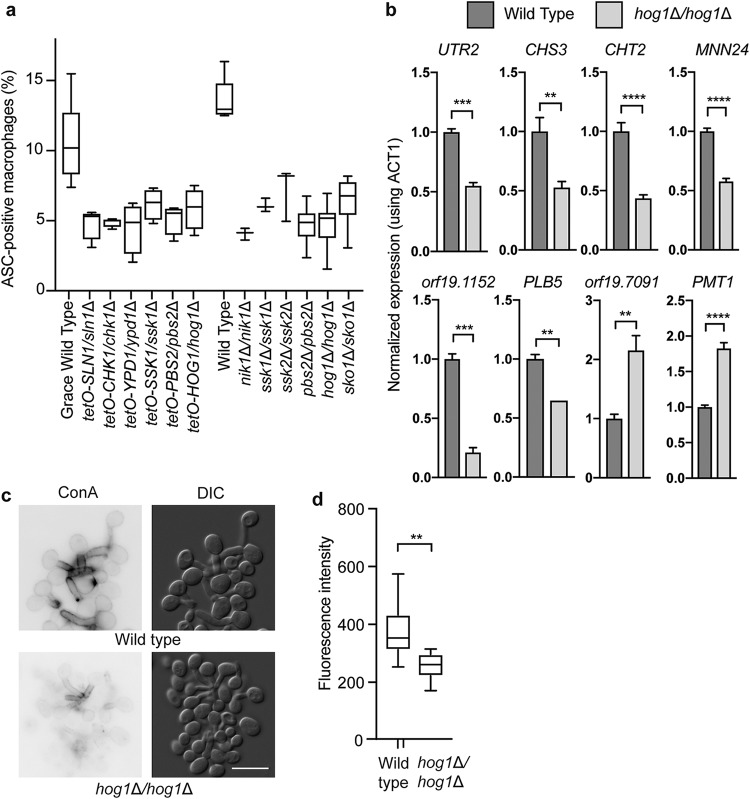
Hog1 signaling is required for induction of macrophage pyroptosis via transcriptional regulation of cell wall genes. (a) Levels of ASC speck formation for mutants of genes in the Hog1 cascade. GRACE strains were incubated with 0.5 µg/ml DOX overnight and during infection to repress target gene expression. Macrophages were infected at an MOI of 1:3. ASC specks were quantified in ASC-mCherry macrophages after 4 h of infection from the average results from two technical replicates from at least two biological replicates. All mutants were significantly (*P* < 0.0001) different from the parent strains, as determined by 1-way ANOVA with Dunnett’s multiple-comparison test. Error bars represent standard deviations. (b) Hog1 is required for transcription of C. albicans genes encoding pyroptosis activators in macrophages. RNA was collected from the indicated strains after incubation with macrophages for 1 h. Significance was determined using 1-way ANOVA. ****, *P* < 0.0001; ***, *P* < 0.005; *, *P* < 0.05. Error bars represent standard errors of the means. (c) Hog1 is required for cell wall remodeling. Cells were incubated with macrophages for 1 h before collection and staining with concanavalin A for mannose. Bar, 10 µm. (d) Quantification of concanavalin A staining after incubation in macrophages for 1 h. Fluorescence levels were quantified using ImageJ from two biological replicates. Significance was determined by unpaired *t* tests. **, *P* < 0.01.

To examine the role of fungal signaling pathways in regulation of host cell pyroptosis, we focused on the p38/Hog1 MAP kinase cascade, as we identified mutants affecting multiple components of this cascade in the pyroptosis screen. In follow-up analysis, we determined that homozygous deletion mutants for six components of this signaling cascade exhibit defective induction of ASC oligomerization in macrophages with no defect in filamentation (Fig. 4a), confirming the results seen with the GRACE mutants. Included in this set were mutants lacking *NIK1*, *SSK2*, and *SKO1*, which are genes that are not included in the GRACE collection. The terminal Sko1 transcription factor of this cascade can also be regulated by the Fun31/Psk1 kinase (39); however, the homozygous transposon mutant of *PSK1* (*psk1*::*TN*/*psk1*::*TN*) was not significantly defective in activation of ASC speck formation (Fig. S3a), suggesting that the major signal is mediated through the Hog1 mitogen-activated protein kinase (MAPK) cascade. Reduced ASC speck formation by strains lacking Hog1 cannot be attributed to reduced phagocytosis, as there was no significant difference between the *hog1*Δ/*hog1*Δ mutant and the wild-type strain (Fig. S3b). Thus, signaling through Hog1 enables C. albicans to respond to the macrophage environment and activate ASC speck formation.

10.1128/mBio.01581-18.3 FIG S3  (a) The Psk1 kinase does not affect pyroptosis. ASC-mCherry macrophages were incubated with the indicated strains at an MOI of 1:3 for 4 h before imaging. Pyroptosis was quantified from two technical replicates from at least two biological replicates. Error bars represent standard deviations. Significance was determined by 1-way ANOVA. (b) The *hog1*Δ/*hog1*Δ mutant strain does not have a defect in pyroptosis. Strains were incubated with J774A.1 macrophages for 30 min before imaging. The numbers of internalized cells were determined from two biological replicates and plotted as the average rate of internalization from at least 10 fields of view. Error bars represent standard deviations. Significance was determined by unpaired *t* tests. (c) Transcripts that did not show Hog1-dependent changes in expression. RNA was collected from the indicated strains after incubation with macrophages for 1 h. Data are from two biological replicates, with three technical replicates each. Error bars represent standard errors of the means. (d) Cell wall staining of the *sko1*Δ/*sko1*Δ mutant shows a decrease in mannose upon entry into macrophages similar to that seen with the wild type. Bar, 20 µm. Error bars represent standard deviations. Significance was determined by unpaired *t* tests. (e) Cell wall staining of the *hog1*Δ/*hog1*Δ mutant shows an increase in chitin upon entry into macrophages compared with the wild type. Bar, 20 µm. Download FIG S3, TIF file, 2 MB. Copyright © 2018 O’Meara et al. 2018 O’Meara et al. This content is distributed under the terms of the Creative Commons Attribution 4.0 International license.

### Hog1 is required for cell wall remodeling in response to macrophage internalization.

Given that Hog1 controls stress-responsive transcriptional programs via the Sko1 transcription factor (39), we examined whether Hog1 might influence pyroptosis by regulating the expression of genes required for pyroptosis. We measured the impact of deletion of *HOG1* on the expression of the 16 genes that had the greatest effect on pyroptosis and contained putative Sko1 binding sites (39–41) in their promoters. Eight of these genes showed Hog1-dependent expression in the macrophage (Fig. 4b; see also Fig. S3c). Many of these encode cell wall proteins or factors that modify the fungal cell wall, suggesting that Hog1-dependent regulation of cell wall factors may be important for inducing pyroptosis. We then examined the levels of cell wall components by fluorescent staining. Using concanavalin A as a measure of mannose levels, we observed significantly higher mannose content in wild-type cells than in the *hog1*Δ/*hog1*Δ mutant cells (Fig. 4c and d). Similarly, the *sko1*Δ/*sko1*Δ mutant also had a defect in mannose levels after incubation in macrophages (Fig. S3d). We also examined chitin levels in response to macrophages by staining with calcofluor white (41). Consistent with previous reports that Hog1 regulates chitin production (41), we saw an increase in chitin content in the *hog1*Δ/*hog1*Δ mutant (Fig. S3e). These experiments suggest that Hog1 signaling is required for appropriate regulation of cell wall remodeling and mannose exposure in response to the macrophage environment.

### Inflammasome activation is required for neutrophil recruitment to lesions during infection.

**FIG 5:**
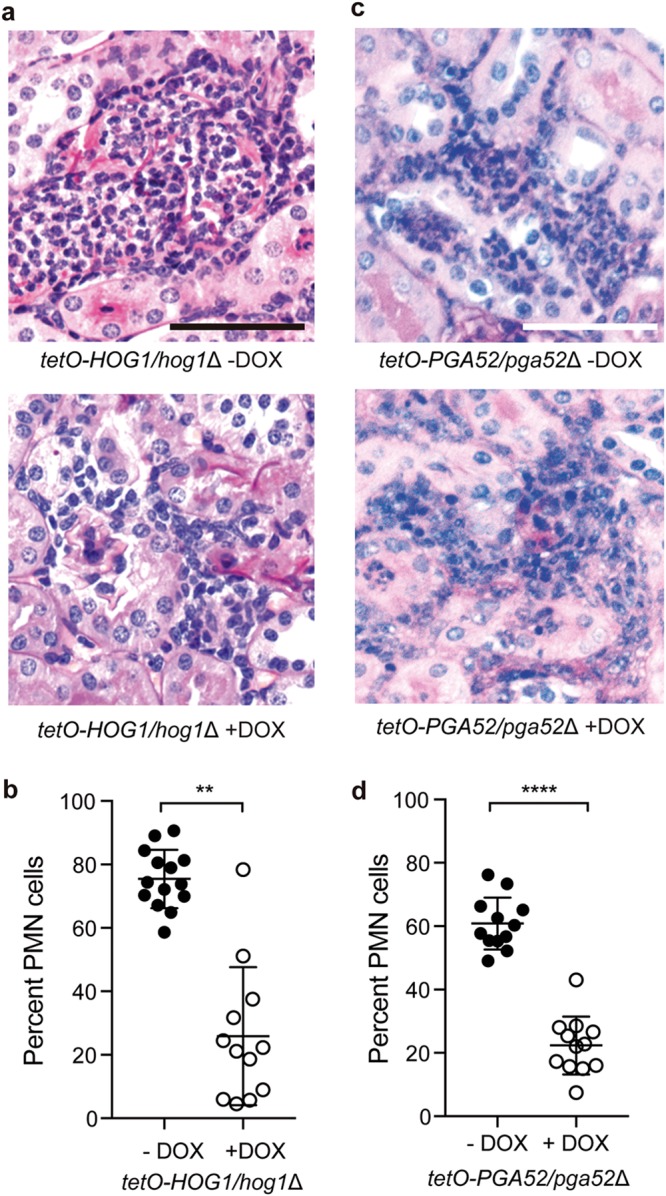
Neutrophil recruitment is influenced by induction of pyroptosis by C. albicans. (a) PAS-treated kidneys from mice infected with the *tetO-HOG1*/*hog1*Δ strain and treated with or without DOX. Bar, 50 µm. (b) Hog1 induces PMN-dominated inflammatory lesions. For each lesion from the PAS-treated kidneys, the percentage of PMN cells was determined by nuclear morphology. *n* = 14 lesions for kidneys without DOX; *n* = 12 lesions for kidneys with DOX. Significance was determined by *t* tests. **, *P* < 0.005. Error bars represent standard deviations. (c) PAS-treated kidneys from mice infected with the *tetO-PGA52*/*pga52*Δ strain and treated with or without DOX. Bar, 50 µm. (d) Pga52 induces PMN-dominated inflammatory lesions. For each lesion from the PAS-treated kidneys, the percentage of PMN cells was determined by nuclear morphology. *n* = 12 lesions for kidneys without DOX; *n* = 12 lesions for kidneys with DOX. Significance was determined by *t* tests. ****, *P* < 0.0001. Error bars represent standard deviations.

The capacity to induce pyroptosis has complex implications for host pathogen interactions. We hypothesized that mutants that are deficient in activating macrophage ASC speck formation would have defects in immune cell recruitment during infection. We examined inflammatory cell influx into the kidney using intravenous models of murine candidiasis. BL/6 mice were given water with DOX or a control preparation (5% sucrose) for 3 days prior to tail vein inoculation with approximately 1 × 10^5^ CFU of the *tetO-HOG1*/*hog1*Δ GRACE strain per mouse. After 24 h, we monitored levels of immune cell infiltrate in the kidneys by staining with periodic acid-Schiff stain (PAS) and levels of kidney fungal burden by plating for CFU. In the absence of DOX, there were multiple foci of inflammatory cells containing mostly PMN cells (Fig. 5a and b). In contrast, in the DOX-treated mice, we observed fewer foci of inflammation, and the lesions were characterized by mononuclear cells (Fig. 5a and b). A potential confounding factor in this analysis was the decrease in fungal burden in the DOX-treated mice (Fig. S4). However, we hypothesized that there would be a distinct profile of immune cell recruitment based on altered induction of macrophage cell death even comparing mice whose fungal burdens were similar. To test this, we examined changes in immune cell infiltrate levels in response to an independent pyroptosis-defective C. albicans strain, the *tetO-PGA52*/*pga52*Δ strain. BL/6 mice were given water with DOX or a control preparation (5% sucrose) for 3 days prior to retro-orbital inoculation with approximately 1 × 10^5^ CFU of the *tetO-PGA52*/*pga52*Δ strain per mouse. After 48 h, we monitored immune cell infiltrate levels in the kidneys using PAS treatment and monitored kidney fungal burden by plating for CFU. Importantly, depletion of Pga52 at that time point did not result in decreased CFU in the kidney (Fig. S4b). Similarly to the Hog1 experiment, we observed foci of inflammation containing mostly PMN cells in the absence of DOX (Fig. 5c and d). When Pga52 was depleted with DOX, we observed that the inflammatory lesions in the infected kidneys were composed primarily of mononuclear cells (Fig. 5c and d), similarly to the results observed with the *tetO-HOG1*/*hog1*Δ strain. These findings are consistent with a role for C. albicans infections in inducing neutrophil recruitment via activation of the inflammasome.

10.1128/mBio.01581-18.4 FIG S4  (a) Hog1 is required for fungal proliferation in the kidneys. Mice (*n* = 6 per group) were given DOX or 5% sucrose in their drinking water for 3 days prior to tail vein injection with the *tetO-HOG1*/*hog1*Δ strain. Kidneys were collected after 24 h, homogenized, and plated for CFU counts. Dots represent data from individual animals. Significance was determined using a *t* test performed on log-transformed data (*P* < 0.0005). Error bars represent standard deviations. (b) Pga52 is not required for fungal proliferation in the kidneys. Mice (*n* = 5 per group) were given DOX or 5% sucrose in their drinking water for 3 days prior to retro-orbital injection with the *tetO-PGA52*/*pga52*Δ strain. Kidneys were collected after 48 h, homogenized, and plated for CFU counts. Dots represent data from individual animals. Error bars represent standard deviations. (c) Pga52 is not required for fungal proliferation in the kidneys. Mice (*n* = 5 per group) were given DOX or 5% sucrose in their drinking water for 3 days prior to retro-orbital injection with the *tetO-PGA52*/*pga52*Δ strain. Kidneys were collected after 24 h, homogenized, and plated for CFU counts. Dots represent data from individual animals. Error bars represent standard deviations. Significance was determined by *t* tests. (d) Pga52 induces PMN-dominated inflammatory lesions. For each lesion from the PAS-treated kidneys, the percentage of PMN cells was determined by nuclear morphology. *n* = 15 lesions for kidneys without DOX; *n* = 18 lesions for kidneys with DOX. Significance was determined by *t* tests. ***, *P* < 0.0005. Error bars represent standard deviations. Download FIG S4, TIF file, 0.8 MB. Copyright © 2018 O’Meara et al. 2018 O’Meara et al. This content is distributed under the terms of the Creative Commons Attribution 4.0 International license.

### C. albicans priming of the NLRP3 inflammasome requires MyD88 and BCL10/MALT1 signaling.

C. albicans engages the NLRP3 inflammasome (6, 10, 33), and previous work suggested that Syk kinase coordinates both priming and activation of the inflammasome (16). However, inhibition of Syk kinase can inhibit phagocytosis of *Candida* species (42, 43), potentially confounding interpretation of these results, as defects in phagocytosis block induction of pyroptosis (Fig. 3) (44). Moreover, the factors required for recognizing internalized fungi and specifically for activating the NLRP3 inflammasome remain largely unknown. To address this, we monitored the impact of lentivirus-encoded short hairpin RNA (shRNA) knockdown of five candidate host factors in THP-1 monocytes on pyroptosis in response to C. albicans. Since depletion of receptors such as dectin-1 result in defects in phagocytosis (45, 46) that would confound our analysis, we focused on inflammasome components and a set of adapters downstream of the major receptors engaged by C. albicans. The shRNA-transduced THP-1 cells were infected with wild-type C. albicans at a multiplicity of infection (MOI) of 1:3 and monitored for host cell death using PI staining.

**FIG 6:**
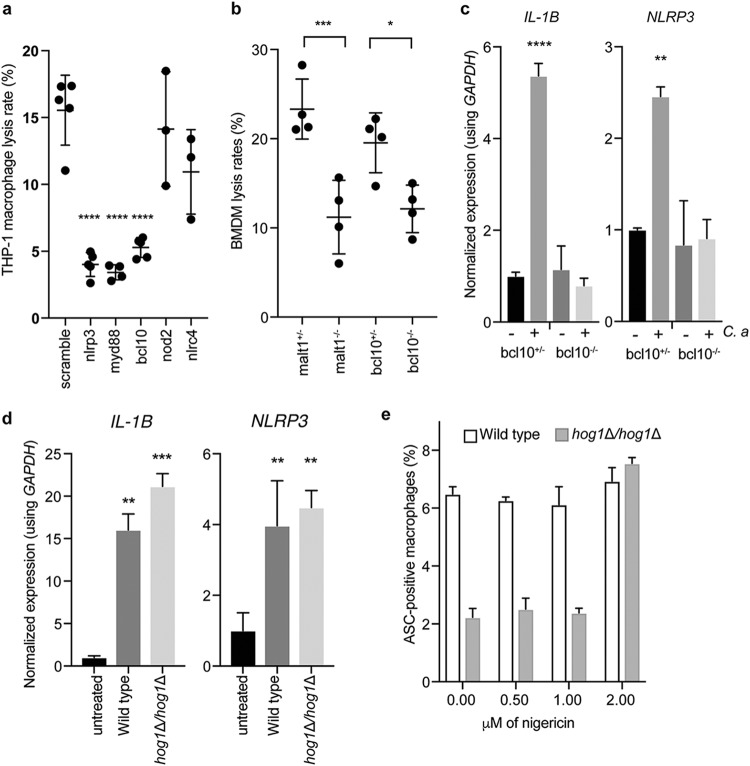
Host cells actively respond to C. albicans infection. (a) Nlrp3, Myd88, and Bcl10 are required for lysis in response to C. albicans. Lentiviral shRNA knockdowns of the indicated genes were performed in THP-1 macrophages, which were then infected with wild-type C. albicans at an MOI of 1:3 for 4 h before imaging for propidium iodide staining was performed. ****, *P* < 0.0001 (one-way ANOVA). Data are combined from two biological replicates. Error bars represent standard deviations. (b) Bcl10 and Malt1 are required for pyroptosis. Bone marrow-derived macrophages were obtained from Bcl10^−/−^ and Malt1^−/−^ knockout mice and their heterozygote littermates. Macrophages were incubated for 4 h with wild-type C. albicans at an MOI of 1:3 before imaging. ***, *P* < 0.005; *, *P* < 0.05 (one-way ANOVAs). Data are combined from two biological replicates. Error bars represent standard deviations. (c) Bcl10 is required for priming the inflammasome. Bone marrow-derived macrophages were obtained from Bcl10^−/−^ knockout mice and their heterozygote littermates. Macrophages were incubated for 3 h with or without C. albicans at an MOI of 1:3 before RNA extraction. ****, *P* < 0.001; **, *P* < 0.01 (one-way ANOVAs). Error bars represent standard errors of the means. (d) IL-1β and NLRP3 transcriptional inductions are not impaired in the *hog1*Δ/*hog1*Δ mutant. RNA was collected from macrophages infected for 3 h at an MOI of 1:3 with the indicated strains. Significance was determined using one-way ANOVA. Data are representative of two biological replicates. Error bars represent standard errors of the means. (e) The *hog1*Δ/*hog1*Δ mutant has a specific defect in inflammasome activation. ASC-mCherry macrophages were incubated with wild-type cells or *hog1*Δ/Δ cells at an MOI of 1:3 for 3 h before the indicated doses of nigericin were added. ASC speck formation was determined after a further 30 min. Error bars represent standard deviations.

As expected, depletion of NLRP3 reduced host cell death, whereas depletion of the intracellular pattern recognition receptor NOD2 (47) or the NLRC4 inflammasome (33) did not have a significant effect on host cell death in response to C. albicans infection (Fig. 6a and S5b). We observed that the MyD88 adapter, which mediates signals downstream of Toll-like receptors (TLRs) and the IL-1 receptor (1), and BCL10, which acts in complex with MALT1 and CARD9 downstream of C-type lectin receptors (CLRs) (1), were also required for host cell death in response to C. albicans (Fig. 6a). To investigate this further, we obtained bone marrow-derived macrophages (BMDMs) from mice lacking BCL10 and MALT1 and from their heterozygote littermates (48, 49). During infection with the C. albicans wild-type strain, both Bcl10^−/−^ and Malt1^−/−^ BMDMs had significantly reduced levels of host cell death compared with BMDMs obtained from the corresponding heterozygous littermates, demonstrating that signaling through Bcl10 and Malt1 is required for host cell death in response to C. albicans (Fig. 6b; see also Fig. S5b). To investigate whether this decrease in host cell death was due to defects in inflammasome priming or activation, we infected Bcl10^−/−^ or Bcl10^+/−^ BMDMs with C. albicans and monitored the induction of *NLRP3* and *IL-1β* transcripts by quantitative reverse transcription-PCR (qRT-PCR). The Bcl10^−/−^ BMDMs were defective in the induction of inflammasome transcripts upon challenge by C. albicans (Fig. 6c), demonstrating that Bcl10 is required for inflammasome priming. Together, these findings suggest that the coordinated activity of both the TLR and CLR pathways is required for priming the inflammasome and inducing pyroptosis in response to C. albicans.

10.1128/mBio.01581-18.5 FIG S5  (a) Representative images of knockdown THP-1 macrophages after 4 h of infection with C. albicans. Bar, 50 µm. (b) Representative images of BMDMs after 4 h of infection with C. albicans. Bar, 50 µm. (c) Inflammasome priming is not affected by *PGA52* depletion. RNA was collected from macrophages infected for 3 h at an MOI of 1:3 with the indicated strains. To deplete target gene expression, the strains were incubated with 0.5 µg/ml DOX overnight and during infection. Significance was determined using one-way ANOVA. Data are representative of two biological replicates. Error bars represent standard deviations. (d) Representative images of ASC-mCherry macrophages after 3 h of infection with the indicated C. albicans strains and 30 min of treatment with 2 µM nigericin. Bar, 50 µm. Download FIG S5, TIF file, 4.8 MB. Copyright © 2018 O’Meara et al. 2018 O’Meara et al. This content is distributed under the terms of the Creative Commons Attribution 4.0 International license.

To determine whether C. albicans mutants with defects in the induction of pyroptosis are specifically defective with respect to priming or activating the inflammasome, we challenged macrophages with wild-type cells or *hog1*Δ/*hog1*Δ cells and measured levels of *NLRP3* and *IL-1β* transcripts. Both strains were able to induce significant increases in *NLRP3* and *IL-1β* expression, indicating that they were not defective in inflammasome priming (Fig. 6d). We observed similar results in challenging macrophages with the *tetO-PGA52*/*pga52*Δ GRACE strain in the absence or presence of DOX (Fig. S5c). We then tested whether the priming induced by the *hog1*Δ/*hog1*Δ strain was functional by adding low doses of the inflammasome activator nigericin to the ASC-FP macrophages and measuring ASC speck formation. At 2 µM nigericin, the defect in *hog1*Δ/*hog1*Δ–induced pyroptosis was fully rescued, suggesting that the impaired capacity of this strain to activate pyroptosis was specific to defects in inflammasome activation (Fig. 6e). There was no effect of nigericin at this dose on uninfected cells (Fig. S5d). Taking the data together, this demonstrates that inflammasome activation and priming can be decoupled in response to C. albicans.

### Macrophage inflammasome activation does not require phagolysosomal leakage.

**FIG 7:**
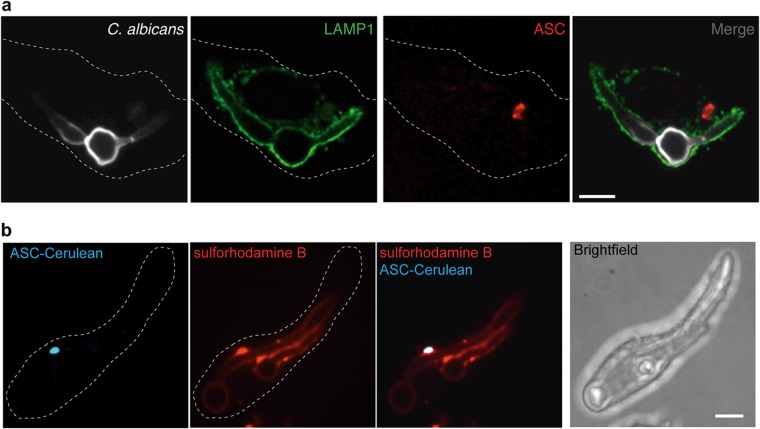
ASC oligomerization does not depend on phagolysosomal rupture during C. albicans infection. (a) Bone marrow-derived macrophages from C57/BL6 mice were infected with wild-type C. albicans for 2.5 h at an MOI of 1:2, fixed with 4% PFA, and immunostained with an anti-Lamp1 antibody to mark the late phagolysosomes, anti-ASC antibody to mark inflammasomes, and calcofluor white to mark C. albicans cells. The images represent one macrophage from two biological replicates. Bar, 10 µm. (b) ASC-cerulean cells were loaded overnight with sulforhodamine B, chased for 1 h with fresh RPMI medium, infected with wild-type C. albicans for 2.5 h at an MOI of 1:2, and then imaged. The images represent one macrophage from two biological replicates. Bar, 10 µm.

Inflammasome activation is usually mediated via cellular intermediary signals, and lysosomal rupture is often associated with induction of pyroptosis (50, 51). To evaluate lysosomal integrity when ASC oligomers form in response to C. albicans infection, we infected bone marrow-derived macrophages with wild-type C. albicans cells for 2.5 h, fixed the cells with 4% paraformaldehyde, and used immunofluorescence to detect endogenous ASC oligomerization and LAMP-1 localization. Using confocal microscopy, we observed C. albicans contained within LAMP-1-associated phagosomes at time points at which ASC oligomerization occurred (Fig. 7a). To validate these findings, we preloaded ASC-cerulean macrophages with sulforhodamine B (*M*_*r*_ = 607) to mark the phagosome and performed live-cell microscopy on infected cells. The dye colocalized with the C. albicans hyphae when ASC specks were present (Fig. 7b; see also Fig. S6), suggesting that there was no leakage from the phagosome, as even transient rupture would allow release of the dye. Thus, C. albicans remains contained within intact phagolysosomes during activation of the cytosolic NLRP3 inflammasome, implicating active host signaling and not merely lysosomal rupture in the activation of inflammasomes in response to internalized C. albicans. We propose that specific defects in fungal cell wall remodeling cause failure to activate the inflammasome, resulting in defects in ASC speck formation and pyroptosis.

10.1128/mBio.01581-18.6 FIG S6  (a) Sulforhodamine B does not stain C. albicans cells. Bar, 10 µm. (b) Sulforhodamine B staining of the phagolysosome disperses upon membrane permeabilization with 0.01% Triton X-100. Bar, 10 µm. (c) Additional image of sulforhodamine B staining of intact phagolysosomes. Bar, 10 µm. (d) Representative image of sulforhodamine B staining of uninfected macrophages. Bar, 10 µm. (e) Additional image of bone marrow-derived macrophages from C57/BL6 mice that were infected with wild-type C. albicans for 2.5 h at an MOI of 1:2. The macrophages were fixed with 4% PFA and immunostained with an anti-Lamp1 antibody to mark the late phagolysosomes, with anti-ASC antibody to mark inflammasomes, and with calcofluor white to mark C. albicans cells. Bar, 10 µm. Download FIG S6, TIF file, 4.6 MB. Copyright © 2018 O’Meara et al. 2018 O’Meara et al. This content is distributed under the terms of the Creative Commons Attribution 4.0 International license.

## DISCUSSION

The ability of a pathogen to cause disease and persist in the human host requires the capacity to evade and circumvent host defense mechanisms. This selective pressure has led to the evolution of diverse mechanisms among microbial pathogens to thwart attack by host immune cells, including the capacity to induce host cell death programs such as macrophage pyroptosis. Here, we performed a systematic analysis of genes that enable the fungal human pathogen C. albicans to sense and respond to the macrophage environment to initiate macrophage pyroptosis. This large-scale genetic analysis of C. albicans induction of host cell death greatly expands on the established set of genes implicated in fungal activation of macrophage pyroptosis and demonstrates that filamentation is dispensable for the induction of pyroptosis in diverse mutant strains. Although our analysis provides the most comprehensive coverage of fungal genes required for responding to mammalian immune cells to date, additional genetic space remains to be explored. Moreover, we focused on pyroptosis, which is only one method by which C. albicans can cause host cell death (10, 11). As one example, the GRACE collection does not contain genes encoding many of the secreted aspartyl proteases that are known to activate IL-1β release in macrophages (20, 52). With the development of additional functional genomic resources in C. albicans and other fungal pathogens, a comprehensive analysis of this host pathogen interface is within reach (53).

The discovery that many of the C. albicans genes required for activation of macrophage pyroptosis are involved in cell wall biosynthesis and regulation is reassuring, given that fungal cell wall remodeling in response to the macrophage phagosome is a crucial initiating event for pyroptosis (24). A simple model is that immune cells exploit the cell wall differences or levels of β-glucan masking between yeast and filaments to sense the relative pathogenicity of this opportunistic pathogen (54). Consistent with this hypothesis, a recent study comparing yeast and hyphae found β-2,3 glucan linkages on filaments that are not present on yeast cells. Our finding that not all mutants with modified β-glucan have a defect in activation of pyroptosis suggests that there may be additional structural features of β-glucan that modify its ability to influence pyroptosis and mask recognition by host cells or that there is redundancy in these biosynthetic cascades. Additionally, we observed that many but not all mannosyltransferases are required for pyroptosis. For example, depletion of the β-mannosyltransferases did not affect pyroptosis. Given that host galectins serve as receptors for β-mannose (55), this suggests that inflammasome activation does not occur through this receptor. As mannans constitute the outermost layer of the C. albicans cell wall, we hypothesize that fungal manipulation of these structures, resulting in either altered masking of β-glucan structures or direct interaction with host receptors, plays a key role in shaping the host cell response. An additional factor to consider is that host glycosidases may be essential for the exposure of the ligand that triggers pyroptosis. Macrophage phagolysosomes contain multiple glycosidases (56) that can act upon fungal mannans, thereby unmasking specific structures for detection by membrane-bound receptors or releasing small sugars that could enter the cytosol and trigger inflammasome activation in a manner analogous to recognition of bacterial peptidoglycan by hexokinase (57). There are clearly multiple possible mechanisms through which cell wall remodeling can trigger host cell death.

Our systematic analysis also implicated C. albicans genes involved in diverse biological processes with no clear link to the fungal cell surface in regulating pyroptosis. For example, transcriptional repression of 11 genes involved in RNA metabolism, including genes involved in mRNA decapping and in snoRNA processing, impaired activation of pyroptosis. This was also the case for genes involved in DNA damage, either directly, as in the case of *RAD3* and *RAD26* (58), or indirectly, such as *FZD1*, *NOC3*, and *ERV29* (59), which are upregulated in response to DNA damage and stress. This link with DNA damage is consistent with the capacity of macrophages to induce C. albicans DNA damage through oxidative stress (60, 61) and suggests that this response may signal entry into phagolysosomes. We also implicated five genes encoding amino acid permeases in activation of pyroptosis. Amino acid sensing and import are important for sensing the macrophage phagolysosome and autoinduction of filamentation through alkalinization (62, 63). Here, we show that individual permeases can influence pyroptosis with a negligible impact on filamentation. Finally, we also implicated the ergosterol biosynthetic gene *ERG5*, consistent with a role for ergosterol in triggering pyroptosis (64). Our results are consistent with the model that diverse strains capable of filamentation in response to macrophages but defective in activation of pyroptosis have an altered cell surface that results in masking or reduction of key triggers.

Diverse environmental cues are sensed by microbial pathogens to produce appropriate cellular responses in different host niches. Our screen identified the Hog1 MAPK signaling cascade as central to enabling fungal cell wall remodeling in response to the macrophage environment. The Hog1 cascade senses osmotic stress, temperature stress, and oxidative stress through the two-component signaling kinases Sln1, Chk1, and Nik1 and transmits the signals to the terminal MAPK, Hog1 (65, 66). We observed that Hog1 is essential for the elaboration of mannan on the fungal cell surface in response to macrophage internalization, likely via Sko1-mediated transcriptional regulation of genes required for cell wall remodeling and pyroptosis.

These findings may have relevance for systemic infections, in that depletion of C. albicans genes that caused decreased pyroptosis rates resulted in altered inflammatory lesions in the kidneys. Although the tradeoffs between fungal growth, immune clearance, and inflammation are complex, we observed a shift away from PMN cell recruitment when mice were infected with C. albicans cells that do not induce pyroptosis, even when the overall fungal burdens were similar, such as in the case of the *tetO-PGA52*/*pga52*Δ conditional expression strain. There are opposing implications of pyroptosis for pathogen and host, as pyroptosis promotes the survival of phagocytosed fungi but also leads to recruitment of neutrophils important for pathogen clearance (18). In addition to its role in the response to engulfment by macrophages, Hog1 also plays important roles in regulating cell wall remodeling in response to neutrophil extracellular traps (67). Potentially, hyphae that are released from pyroptotic macrophages could have altered interactions with other immune cells, thus providing an additional layer of complexity to the host-pathogen interface.

The capacity of the host to effectively respond to invading pathogens also requires active signaling. We determined that both MyD88 and BCL10/MALT1 are required for macrophage pyroptosis in response to C. albicans infection and that neither TLR signaling nor CLR signaling alone is sufficient to allow pyroptosis. Mice deficient in MyD88 are susceptible to systemic candidiasis (68, 69), and MyD88^−/−^ BMDMs are defective in killing intracellular C. albicans (70), similarly to results seen in mice lacking NLRP3 (16). In dendritic cells, CARD9, Vav proteins, and Dectin-1 can activate the BCL10-MALT1 pathway (71, 72). Our results demonstrate that the BCL10-MALT1 pathway is required for priming the inflammasome for host programed cell death. Using specific C. albicans mutants, we demonstrate that inflammasome priming and activation can be decoupled, in contrast to previous work that suggested that the Syk kinase coordinates both of these processes (16). This implies that macrophages may respond to pathogenic C. albicans cells by activating inflammasomes in response to specific signals. We propose that altered cell surfaces are required for this activation, as lysosomal rupture and C. albicans filamentation are dispensable for this process.

The host-pathogen interface offers a promising target for much-needed novel therapeutics to treat infectious disease. Targeting pathogen factors that enable disease expands the conventional target space for proteins that are essential for pathogen survival (73). Targeting virulence factors such as morphogenesis may provide a viable strategy for reducing disease caused by C. albicans (74). An alternative approach involves targeting host factors that modulate disease progression. In this context, treatment with an NLRP3 inhibitor in an experimental model of vulvovaginal candidiasis reduces the ineffective and pathological inflammatory response without altering pathogen abundance (20). By elucidating the circuitry controlling fungal activation of host programed cell death, we have identified mechanisms governing a key virulence trait in a leading fungal human pathogen.

## MATERIALS AND METHODS

### Strains and culture conditions.

All strains were maintained in cryo-culture at −80°C using 25% glycerol and passaged in yeast extract-peptone-dextrose (YPD). Individual strains are listed in Table S3 in the supplemental material.

10.1128/mBio.01581-18.9 TABLE S3  Strains used in this study. Download TABLE S3, XLSX file, 0.04 MB. Copyright © 2018 O’Meara et al. 2018 O’Meara et al. This content is distributed under the terms of the Creative Commons Attribution 4.0 International license.

### High-throughput imaging.

J774A.1 macrophages were diluted to 5 × 10^5^ cells/ml in RPMI medium–3% heat-inactivated fetal bovine serum (HI-FBS) (Gibco), and 100 µl was added to 96-well plates (Sarstadt) and incubated at 37°C with 5% CO_2_ for 18 h. C. albicans GRACE strains were inoculated into 100 µl of YPD–0.05 µg/ml DOX in 96-well plates and incubated at 30°C. This dose of DOX is sufficient to repress gene expression in rich medium (24). The following day, 50 µl of RPMI medium–0.15 µg/ml DOX–3 ng/ml propidium iodide was added to each well of macrophages for a final concentration of 0.05 µg/ml DOX. The GRACE strains were diluted 1:10 into YPD with 0.05 µg/ml DOX, and the OD_600_ was determined using a spectrophotometer. Then, 10 µl of the diluted C. albicans cells was added to the macrophages and coincubated for 3 h at 37°C with 5% CO_2_. Each well was then imaged on a SpectraMax i3 imager (Molecular Devices) using transmitted light to determine the extent of filamentation and the red fluorescent protein (RFP) channel for propidium iodide foci to quantify macrophage lysis. The number of propidium iodide foci per well was determined using SpectraMax software (Molecular Devices).

For each experiment, a dilution series of the GRACE parental strain was used to infect macrophages, and the amount of PI foci in response to each dilution was used to build a model for the expected lysis rates normalized to the number of cells used for infection. Each wild-type model is fitted by calling the stats::loess [formula = log (PI.counts) ~ OD, data=wt] function in R, with the shaded region representing the 95% confidence interval under an assumed t-distribution of errors and estimated effective degrees of freedom.

The transmitted light images were scored for degree of filamentation, where 0 represents yeast and 3 represents full filamentation. At least two fields of view for each infection were scored, and any strains with discrepancies in scores between fields were retested individually.

### C. albicans survival in macrophages.

C. albicans survival during interaction with macrophages was determined as previously described (75). Briefly, macrophages were prepared as described above and were infected with a serial dilution of C. albicans cells in RPMI medium–3% FBS, with or without 40 nM KCl. The assay was performed with 8 technical replicates per condition, in biological triplicate. After 24 h at 37°C in 5% CO_2_, microcolonies were counted using an inverted microscope. Statistically significant differences between treated and untreated macrophages were determined using unpaired *t* tests in Prism 7 (GraphPad).

### ASC oligomerization assay.

Immortalized bone marrow-derived macrophages harboring an ASC-mCherry fusion protein (gift from Eicke Latz) were diluted to 6 × 10^5^ cells/ml in RPMI medium–3% FBS, and 100 µl was used to seed each well of a 96-well plate. C. albicans GRACE strains were incubated overnight in 100 µl of YPD with 0.5 µg/ml DOX and diluted to 5 × 10^6^ cells/ml in YPD with 1.5 µg/ml DOX, and 50 µl was used to infect the ASC mCherry macrophages in duplicate. C. albicans homozygous deletion strains were incubated overnight in 100 µl of YPD and diluted to 5 × 10^6^ cells/ml in YPD, and 50 µl was used to infect the ASC mCherry macrophages in duplicate. At 4 h postinfection, the wells were imaged on an AxioVision inverted microscope (Zeiss) at 10× magnification on the differential interference contrast (DIC) and tetramethyl rhodamine isocyanate (TRITC) channels, with at least two images per well. ASC oligomerization events in macrophages with internalized C. albicans cells were quantified using ImageJ, with at least 500 infected macrophages counted per strain, in at least two biological replicates. Statistically significant differences between wild-type and mutant strains were determined using one-way analyses of variance (ANOVAs) in Prism 7 with Dunnett’s multiple-comparison test. Each strain was compared with the appropriate parental wild-type strain.

### C. albicans RNA extraction.

J774A.1 macrophages were diluted to 5 × 10^5^ cells/ml in RPMI medium–3% FBS, and 10 ml was used to seed a T75 tissue culture flask. C. albicans cells were incubated overnight in YPD and diluted to 1 × 10^7^ cells, and 1 ml was used to infect the macrophages. After 1 h, the samples were washed with phosphate-buffered saline (PBS), and the internalized C. albicans cells were collected by lysing the macrophages with 0.1% SDS, washing the samples twice with cold water and subjecting them to vortex mixing, and snap-freezing the C. albicans cell pellets. RNA was extracted using an RNeasy plant minikit (Qiagen), and cDNA was amplified by the use of an Ambion cDNA synthesis kit and the provided random primers.

### Macrophage RNA extraction.

Macrophages were diluted to 5 × 10^5^ cells/ml in RPMI medium–3% FBS, and 2 ml was used to seed a 6-well tissue culture plate (Sarstedt). C. albicans cells were incubated overnight in YPD and diluted to 1 × 10^6^ cells, and 1 ml was used to infect the macrophages. After 3 h, the samples were washed with PBS, and cells were collected using a cell scraper. Cells were washed once in PBS before being snap-frozen. RNA was extracted using QiaShredder columns and AN RNeasy kit (Qiagen), and cDNA was amplified from 2 µg of RNA using an Ambion cDNA synthesis kit and the provided random primers.

### qRT-PCR.

PCR was performed using Fast SYBR green master mix (Applied Biosystems) and a Bio-Rad CFX384 real-time system, under the following cycling conditions: 95°C for 3 min, followed by 95°C for 10 s and 60°C for 30 s, for 40 cycles. Reactions were performed in triplicate for two biological replicates. Data were analyzed using Bio-Rad CFX Manager 3.1. All data were normalized to the *ACT1* or *GPD1* reference genes for C. albicans RNA and to the *GAPDH* gene for mouse RNA. Error bars show the standard errors of the means. Significance was determined using unpaired two-tailed *t* tests in Prism 7 (GraphPad). Primer pairs and sequences are listed in Table S4.

10.1128/mBio.01581-18.10 TABLE S4  Oligonucleotides used in this study. Download TABLE S4, XLSX file, 0.01 MB. Copyright © 2018 O’Meara et al. 2018 O’Meara et al. This content is distributed under the terms of the Creative Commons Attribution 4.0 International license.

### Phagocytosis assay.

J774A.1 macrophages were seeded at a density of 1 × 10^6^ cells/ml onto coverslips in 24-well plates. The C. albicans cells were incubated overnight and then diluted to a 3 × 10^6^ cells/ml into RPMI medium–3% FBS and used to infect the macrophages at an MOI of 1:3. Cocultures were spun for 1 min at 1,000 rpm to synchronize phagocytosis. After 30 min, the medium was removed and the cells were fixed with 4% paraformaldehyde (PFA) for 10 min. The cells were washed three times in PBS, blocked for 10 min in 0.4% bovine serum albumin (BSA), and stained with anti-Candida antibody (Abcam, Inc.; catalog no. ab53891) (1:200 dilution) for 1 h at room temperature. The primary antibody was removed, and coverslips were washed three times in PBS and then incubated with an anti-rabbit Alexa 488 antibody for 1 h. The secondary antibody was removed, and coverslips were washed three times in PBS. The macrophages were then incubated with 0.4% Triton for 15 min, blocked in 0.4% BSA for 10 min, and incubated with the anti-*Candida* antibody (1:200 dilution) for 1 h. The primary antibody was removed, and the coverslips were washed three times in PBS and then incubated with anti-rabbit Alexa 594 antibody for 1 h. The slides were then washed three times with PBS and visualized on an AxioVision Observer imaging system. Statistically significant differences between wild-type and mutant strains were determined using one-way ANOVAs in Prism 7 (GraphPad), with Dunnett’s multiple-comparison test.

### Cell surface staining.

C. albicans cells were collected as described for RNA extraction. Instead of snap-freezing, cells were incubated with 100 µg/ml concanavalin A conjugated to fluorescein isothiocyanate (FITC) and 5 µg/ml of calcofluor white for 2 min, washed with PBS, and then imaged using an AxioVision Observer imaging system on the green fluorescent protein (GFP) channel for mannose and the DAPI (4′,6-diamidino-2-phenylindole) channel for chitin. To quantify fluorescence intensity, the fluorescence of each image was normalized to the cell area using ImageJ. At least five fields were imaged for each strain, in at least two biological replicates. Significance was determined using unpaired two-tailed *t* tests in Prism 7 (GraphPad).

### MIC assays and checkerboards.

Drug tolerance assays were performed in flat-bottom, 96-well microtiter plates (Sarstedt) using a modified broth microdilution protocol. For target gene depletion in the GRACE *tetO* strains, cells were incubated overnight in 0.5 µg/ml DOX before being assayed for drug sensitivity in the presence of 0.5 µg/ml DOX. MIC tests were set up in a total volume of 0.2 ml/well, with 2-fold dilutions of each drug in YPD. Plates were incubated in the dark at 30°C before OD_600_ values were determined using a spectrophotometer (Molecular Devices). Each strain was tested in technical and biological replicates. MIC data were quantitatively displayed with color using the program Java TreeView 1.1.1 (http://jtreeview.sourceforge.net/).

### Animal experiments.

All experiments were performed in accordance with protocols approved by the University of Rochester University Committee on Animal Research or the University of San Francisco Committee on Animal Research. Female BL/6 mice (*n* = 6 per group for the *tetO-HOG1*/*hog1*Δ experiments; *n* = 5 per group for the *tetO-PGA52*/*pga52*Δ experiments) were treated with drinking water containing 2 mg/ml doxycycline or with a control preparation (5% sucrose) for 3 days prior to inoculation. Mice were infected via tail vein with approximately 5 × 10^5^ CFU of the *tetO-HOG1*/*hog1*Δ GRACE strain and via retro-orbital infection with approximately 1 × 10^5^ CFU of the *tetO-PGA52*/*pga52*Δ GRACE strain. After 24 h, the animals were sacrificed and kidneys were harvested for fungal burden and histology using previously reported methods (22). PAS-treated slides were used to assess cellular inflammation using samples from kidneys. Percentages of PMN cells were determined by nuclear morphology in the histology micrographs. At least 12 lesions were counted per treatment, with at least 50 immune cells counted per field of view. The kidney fungal burdens were determined by plating on YPD with vancomycin and gentamicin. The log-transformed fungal burden data for the DOX groups and no-DOX groups were compared using *t* tests. The reported fungal burden data from the *tetO-HOG1*/*hog1*Δ experiment are from one of two replicate experiments that gave essentially identical results. The reported fungal burden data from the *tetO-PGA52*/*pga52*Δ experiment are from one replicate. A second replicate was performed, and the fungal burden was assessed at 24 h; at that time point, there was a small but significant increase in fungal burden (*P* = 0.0308) (see Fig. S5 in the supplemental material).

### Phagolysosome imaging.

Bone marrow-derived macrophages from C57/BL6 mice were incubated overnight on glass coverslips. Macrophages were infected with wild-type C. albicans at an MOI of 1:2 for 2.5 h. The cells were then fixed with 4% paraformaldehyde, washed 3 times in PBS, and then permeabilized and blocked by incubation in PBS–0.01% Triton X-100 (PBS-T)–2.5% milk for 30 min at room temperature. The cells were then stained with 1 µg/ml calcofluor white to label C. albicans, a 1:20 dilution of anti-Lamp1 antibody (catalog no.1D4B-s; Developmental Studies Hybridoma Bank), and a 1:100 dilution of anti-ASC antibody–PBS-T–2.5% milk. To visualize the Lamp1 antibody, the cells were incubated with an Alexa 647-conjugated anti-rat secondary antibody (Jackson ImmunoResearch Labs) (1:1,000 dilution). The immunostained cells were imaged on an Axiovert 200M microscope (Zeiss) and a spinning-disk confocal microscope (Yokogawa CSU-X1; Quorum Technologies), using Volocity software. For sulforhodamine B imaging, ASC-cerulean cells were loaded overnight with sulforhodamine B, chased for 1 h with fresh RPMI medium, infected for 2.5 h in RPMI medium–3% FBS at an MOI of 1:2, and then imaged on an Axiovert 200M microscope (Zeiss) spinning-disk confocal microscope.

### shRNA knockdown.

Knockdowns were performed as previously described (76). Briefly, THP-1 monocytes were infected with lentivirus-encoded shRNAs by spinoculation. shRNAs were created in pLKO.1-based lentiviral particles (76) and tested for silencing of the target gene using real-time qPCR. Cells were infected at equal titers in medium containing 8 µg/ml Polybrene and selected after 24 h with 2 µg/ml puromycin. THP-1 human monocyte cells were differentiated into macrophages with 50 ng/ml phorbol myristate acetate (PMA) for 48 h, followed by a 24-h rest period prior to infection.

### BMDM culture.

Bone marrow-derived macrophages (BMDM) were prepared by harvesting bone marrow from femurs and tibias of the relevant mouse strain (48, 49). The mice were 8 to 10 weeks old. BCL10 mice were both females and littermates/cage mates. MALT1 mice were both males and also littermates/cage mates. Mice were housed under specific-pathogen-free conditions. All procedures were approved by the Animal Ethics Review Committee of the University of Toronto, which is subject to the ethical and legal requirements under the province of Ontario’s Animals for Research Act and the Federal Council on Animal Care. Mice were euthanized by CO_2_ inhalation followed by cervical dislocation. Femurs and tibias were removed, and bone marrow was isolated. Cells were cultured in conditioned Dulbecco’s modified Eagle medium (DMEM) for 7 days with 10% fetal bovine serum (FBS), 1% GlutaMAX, 1% penicillin-streptomycin, 10 mM HEPES, and 20% 3T3-conditioned media containing macrophage colony-stimulating factor (M-CSF) in 10-cm-diameter plates (77). Cells received an additional 10 ml of conditioned media on day 3, and nonadherent cells were removed by gentle washing on day 7. BMDMs were collected and seeded at 1 × 10^5^ cells/ml and allowed to adhere overnight before use.
