# Supplementary figures and images for: High-Throughput Screening Identifies Genes Required for Candida albicans Induction of Macrophage Pyroptosis
Source: mBio. 2018 Aug 21;9(4):e01581-18. doi: 10.1128/mBio.01581-18 (PMC6106084; doi:10.1128/mBio.01581-18)

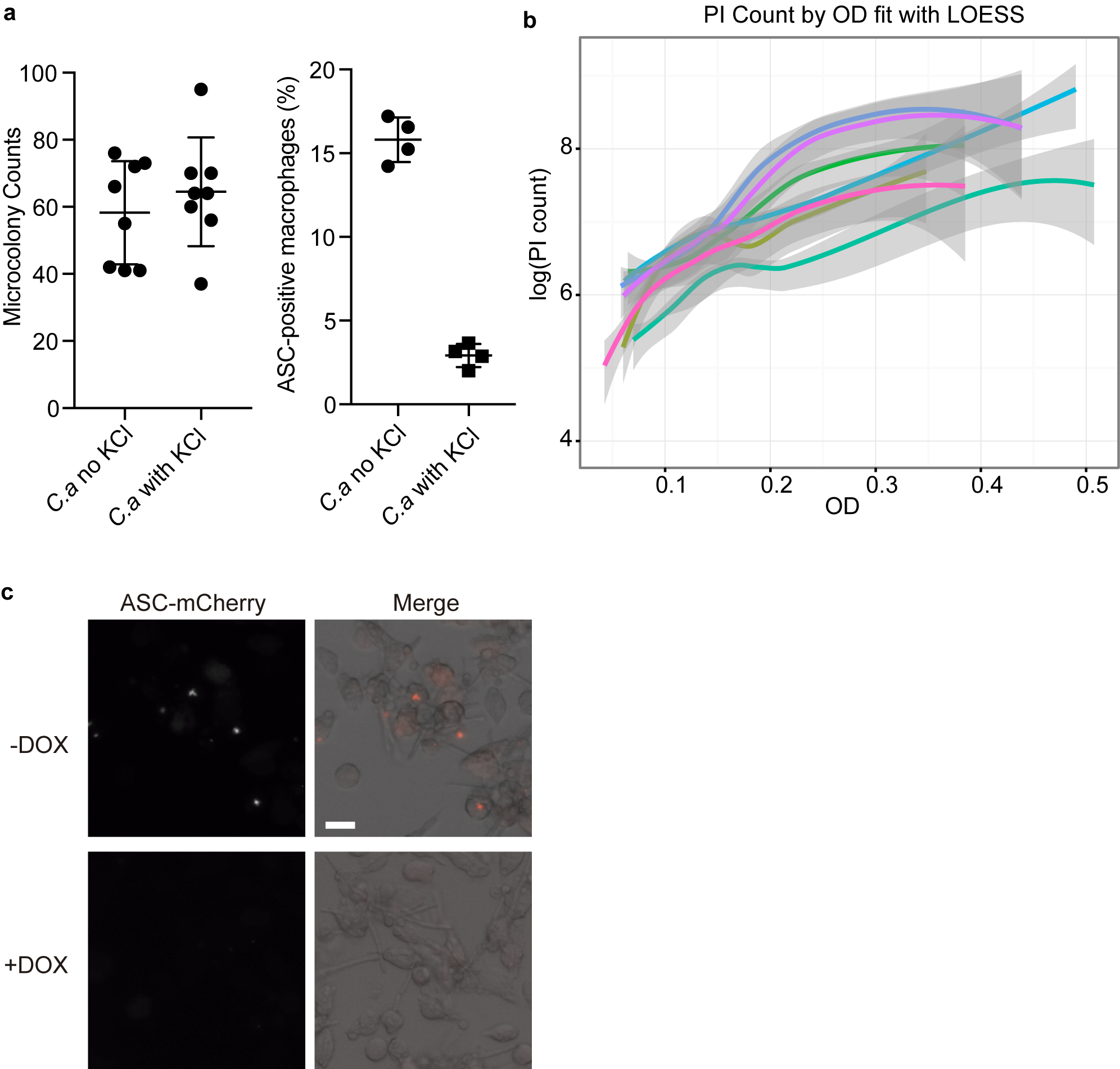

Supplement: FIG S1 [file mbo004184033sf1.tif]

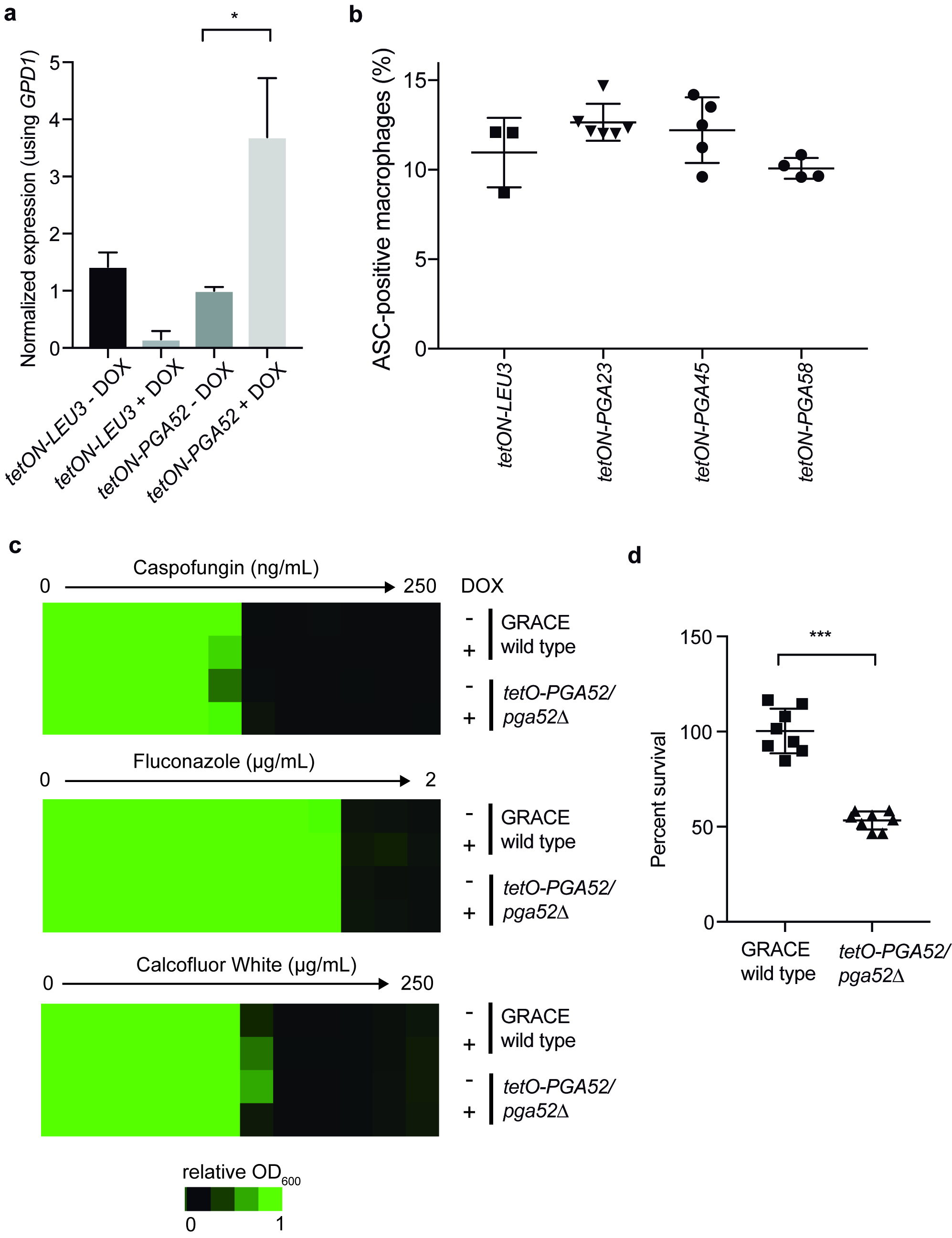

Supplement: FIG S2 [file mbo004184033sf2.tif]

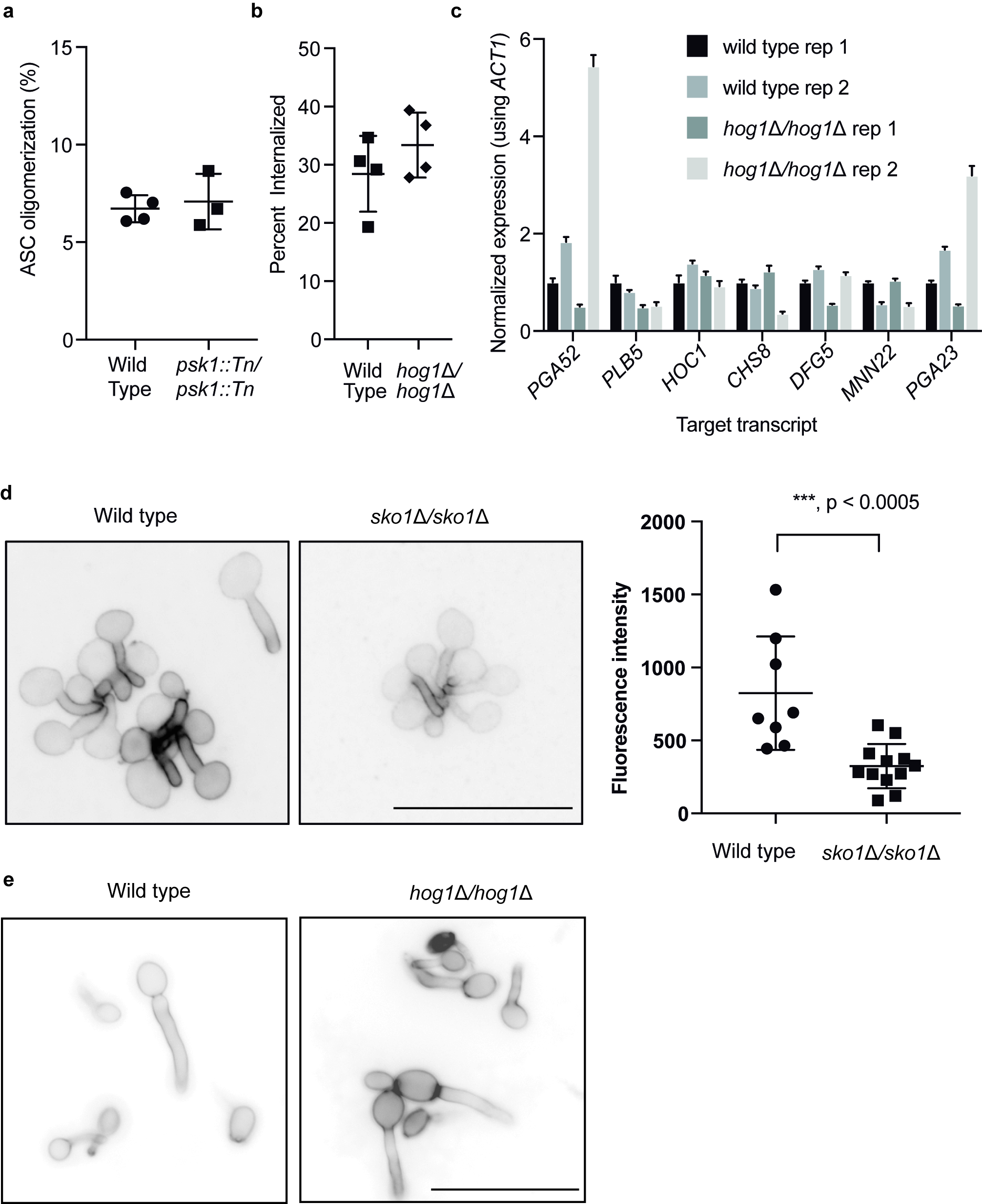

Supplement: FIG S3 [file mbo004184033sf3.tif]

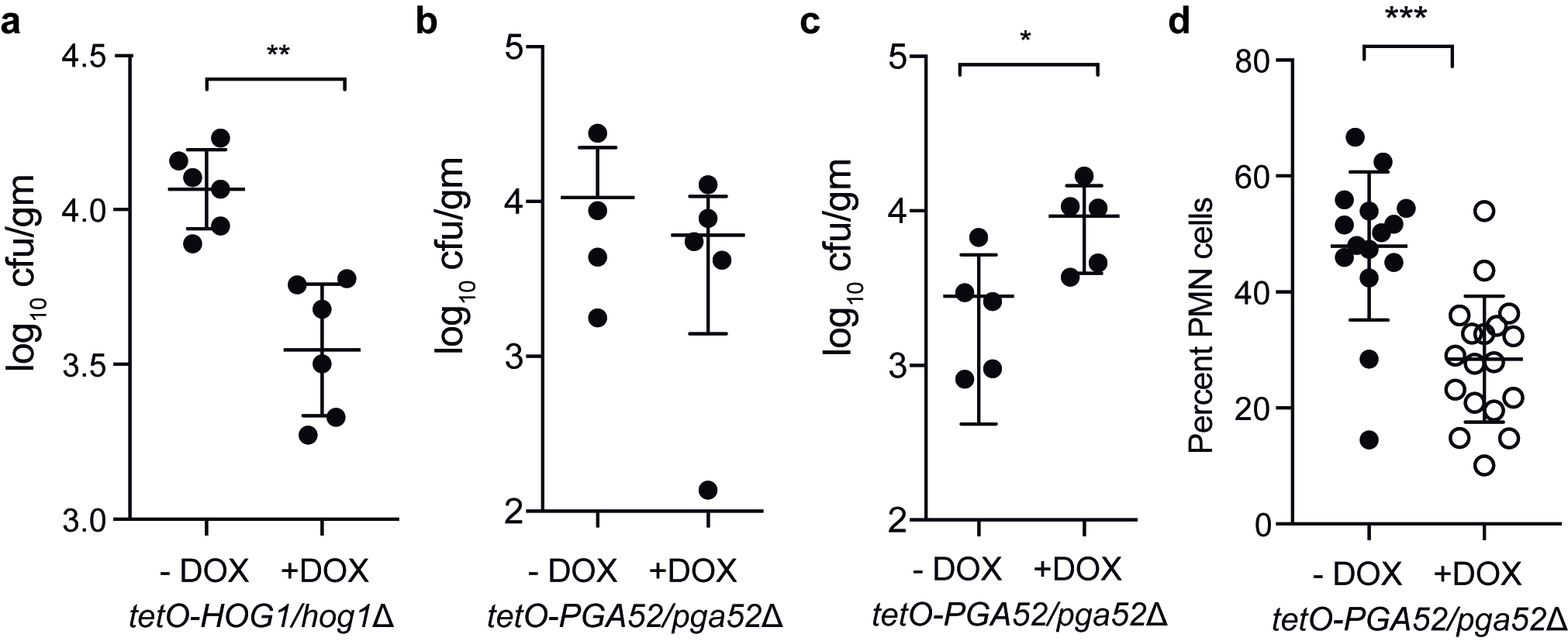

Supplement: FIG S4 [file mbo004184033sf4.tif]

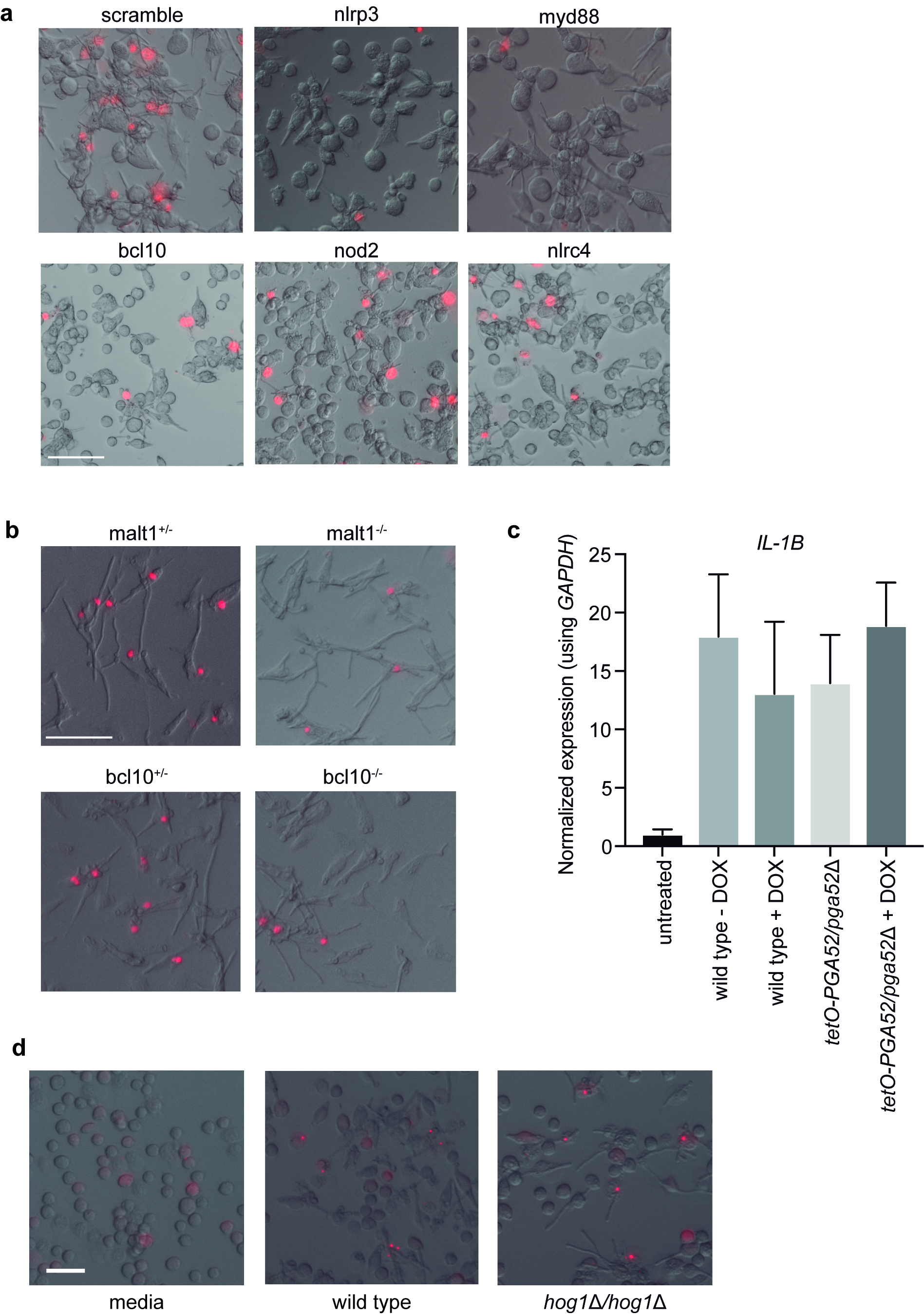

Supplement: FIG S5 [file mbo004184033sf5.tif]

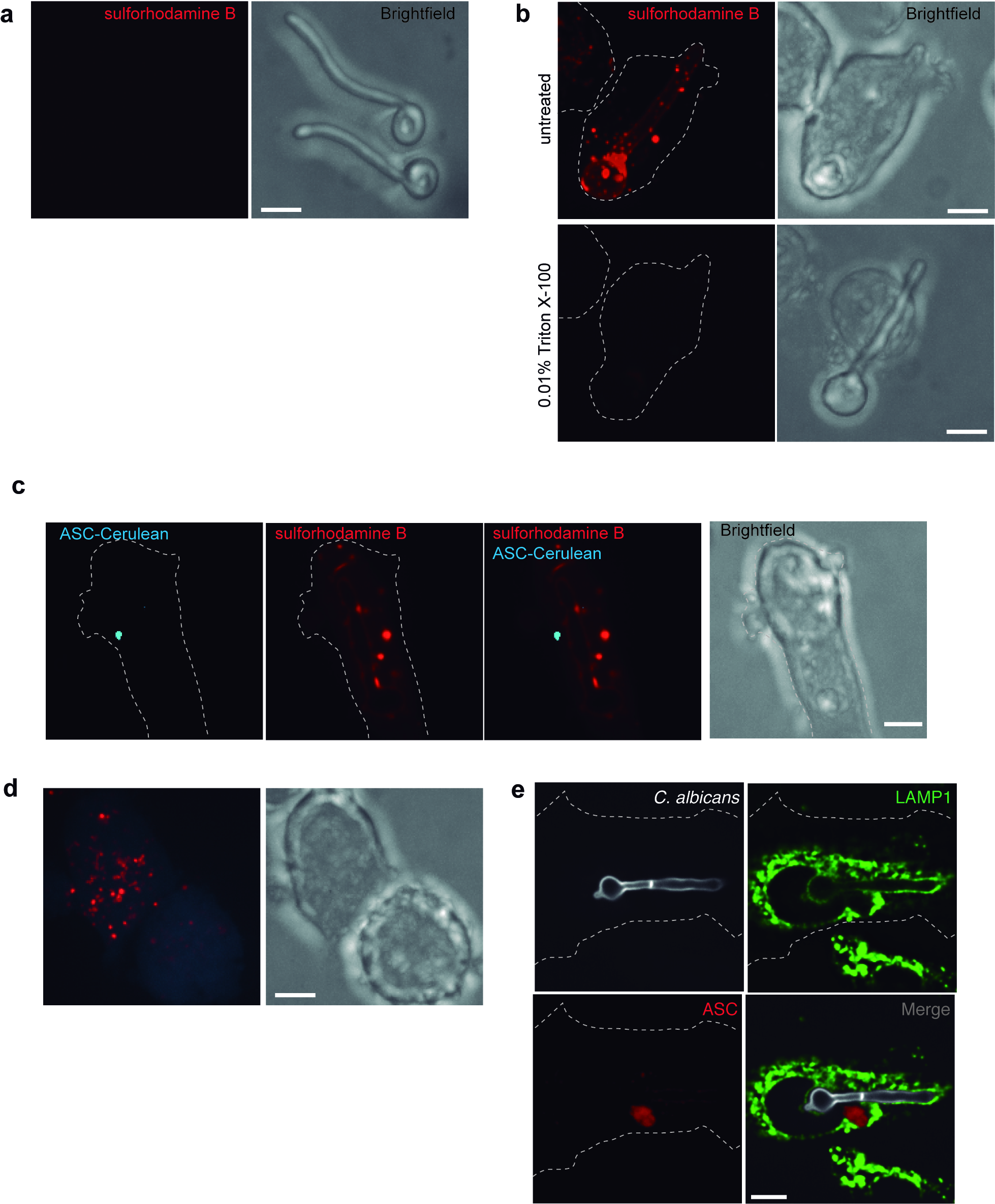

Supplement: FIG S6 [file mbo004184033sf6.tif]
